# Comparison of breast cancer survival in two populations: Ardabil, Iran and British Columbia, Canada

**DOI:** 10.1186/1471-2407-9-381

**Published:** 2009-10-28

**Authors:** Alireza Sadjadi, T Gregory Hislop, Chris Bajdik, Morteza Bashash, Anahita Ghorbani, Mehdi Nouraie, Masoud Babaei, Reza Malekzadeh, Parvin Yavari

**Affiliations:** 1 Digestive Disease Research Center, Shariati Hospital, Tehran University of Medical Sciences; Kargar Street, Tehran, Iran; 2 Department of Epidemiology, University Medical Center Groningen, Groningen, the Netherlands; 3 Cancer Control Research Program, BC Cancer Agency, Vancouver British Colombia, Canada; 4 Department of Health and Community Medicine, School of Medicine and Department of Epidemiology, School of Public Health, Shahid Behshti University of Medical Sciences, Tehran Iran

## Abstract

**Background:**

Patterns in survival can provide information about the burden and severity of cancer, help uncover gaps in systemic policy and program delivery, and support the planning of enhanced cancer control systems. The aim of this paper is to describe the one-year survival rates for breast cancer in two populations using population-based cancer registries: Ardabil, Iran, and British Columbia (BC), Canada.

**Methods:**

All newly diagnosed cases of female breast cancer were identified in the Ardabil cancer registry from 2003 to 2005 and the BC cancer registry for 2003. The International Classification of Disease for Oncology (ICDO) was used for coding cancer morphology and topography. Survival time was determined from cancer diagnosis to death. Age-specific one-year survival rates, relative survival rates and weighted standard errors were calculated using life-tables for each country.

**Results:**

Breast cancer patients in BC had greater one-year survival rates than patients in Ardabil overall and for each age group under 60.

**Conclusion:**

These findings support the need for breast cancer screening programs (including regular clinical breast examinations and mammography), public education and awareness regarding early detection of breast cancer, and education of health care providers.

## Background

Despite the extensive knowledge about incidence and survival rates for cancer in the western world, little information is available for the majority of developing countries [1,2]. International comparisons involving developing countries are few in number. Where done, survival differences have been largely attributed to differences in patient's age, stage of disease at diagnosis, and the presence of metastasis. Socioeconomic factors, differential access to health care, insurance status, comorbidities, and tolerance to prescribed treament have also been suggested to determine survival [3-5]. Immigration status and ethnicity may also play a role. A study of breast cancer among ethnic Chinese women reported that those born in East Asia had lower survival than those born in the US [6]. A recent study in British Columbia (BC) compared survival for three cancer sites in Chinese, South Asians and the predominantly Caucasian general population and found that Chinese women had the highest survival rates for both breast and cervical cancer, whereas South Asian women had the highest rate for colorectal cancer and the lowest rate for cervical cancer [7].

Patterns in cancer incidence can provide important insight into the impact of lifestyle upon cancer development whereas patterns in survival can provide information about the burden and severity of cancer. Identifying differences in survival between populations can help to uncover gaps in systemic policy and program delivery, and support the planning of enhanced cancer control systems [7]. The aim of this study was to describe one-year survival rates for breast cancer in two populations using population-based cancer registries: Ardabil, Iran, and BC, Canada.

## Methods

### Study groups

Two groups with established population-based cancer registries were examined: residents of Ardabil, Iran and BC, Canada.

Ardabil province, located in the northwestern Iran, is a mountainous land with an area of nearly 18,000 square kilometers and a population of 1.1 million persons, 46% living in urban areas. The Ardabil Cancer Registry (ACR) was established in 2003 by the Digestive Diseases Research Center (DDRC) of Tehran University of Medical Sciences and Ardabil University of Medical Sciences (ARUMS), with collaboration of the International Agency for Research on Cancer (IARC). There are 4 kinds of information collected by the registry: patient demographics, tumor characteristics, treatment and patient outcome. Data are actively collected for newly-diagnosed cancer cases among permanent residents of Ardabil province. Patients who are diagnosed in other provinces are captured in the ACR through sharing of data among Iran's provinces. All rural residents are covered by a family physician network and have governmental medical insurance. Reporting cases of cancer to the ACR is obligatory for family physicians. Ardabil's cancer patterns have been studied since the 1970s [8], its cancer registry is relatively complete, its population is largely homogeneous (98% being of Azeri ethnicity), and there is minimal immigration into this area [9].

In contrast, BC, the westernmost province in Canada, has a land area of nearly 945,000 square kilometers and a population of about 4 million persons with various ethnic backgrounds [10]. The provincial cancer registry was established in 1969, with cancer registration mandated by law. It has excellent standards of quality control, completeness of registration and follow-up. It contains personal and demographic information as well as information on diagnosis and death of all the cases of cancer diagnosed among BC residents (http://www.bccancer.bc.ca/HPI/CancerStatistics accessed May 8, 2009). For BC, there is universal health care and the majority of the population has geographically accessible cancer treatment.

### Data collection

#### Study population

The International Classification of Disease for Oncology (ICDO) was used for coding cancer morphology and topography [11]. All newly diagnosed cases of breast cancer (ICDO C50.0-50.9) in women 20 years of age and older were identified in the Ardabil cancer registry for the period 2003-2005 and the BC cancer registry for the year 2003. A 3-year period was used in Ardabil because there are few cases of breast cancer in a single year and statistical results would be unstable.

Survival time was then determined from cancer diagnosis to death. In Ardabil province, information on survival status and the date of death (if deceased) was collected directly by interviewing the registered cases or their families. In addition, the death registry in Ardabil was used to confirm the collected information and to gather this data for registered cases who could not be contacted for interview. Information on the patient's age and date at diagnosis, gender and cancer site was obtained from the cancer registry.

In BC, the survival status of all registered cancer patients is routinely collected by the cancer registry from government vital statistics. Information on the patient's age and date at diagnosis, gender, cancer site, survival status and date of death (if deceased) was collected directly from the cancer registry.

#### Analysis

Adults were defined as people age 20 years and older. Age-specific one-year survival rates were calculated for adult women in the age groups 20-39 years, 40-49 years, 50-59 years and 60+ years. The relative survival rate [12] and a weighted standard error (SE) were calculated using life-tables for each country.

## Results

Table 1 shows the number of adult women diagnosed with breast cancer during the study period in Ardabil and BC, and a one-year survival rate for each age group. Breast cancer patients in BC had greater one-year survival rates than patients in Ardabil overall and for each age group under 60. The median age of breast cancer diagnosis was 61 years (range 24-104 years) in BC and 44 years (range 21-86 years) in Ardabil. About 23% of BC patients and 64% of Ardabil patients were younger than age 50 at the time of diagnosis. The age-standardized one-year relative survival rates in BC was 0.99 (SE = 0.004) for adult women younger than age 50 years and 0.97 (SE = 0.012) for women age 50 and older. The age-standardized 1-year relative survival rate in Ardabil was 0.92 (SE = 0.020) for adult women younger than age 50 and 0.95 (SE = 0.037) for women age 50 and older.

**Table 1:** One -year survival rates for female invasive breast cancer cases diagnosed in 2003 in Ardabil (Iran) and British Columbia (Canada) by age group.

	Ardabil		British Columbia	
**Age**	**Number of Cases**	**One - year Survival rate**	**Number of Cases**	**One -year Survival rate**

20-39	39	0.92	124	0.99

40-49	36	0.92	430	0.99

50-59	27	0.89	597	0.97

60+	16	1.00	1290	0.92

Total	118	0.92	2441	0.95

## Discussion

Patterns of cancer incidence and survival vary around the globe and demographic, ecologic, environmental, cultural, and genetic variables may all contribute to this heterogeneity. This first study comparing breast cancer survival between Iran and BC may contribute to this understanding. There are clear differences in survival between these two populations which reflect variations in severity of cancer, possibly resulting from policy and practices regarding screening and treatment, cancer biology, and cancer registration.

Stage of the disease is an important determinant of survival in patients with breast cancer, but stage is unavailable in both the Ardabil and BC registries. Based on experience, we estimate that about 30% of Ardabil patients and 70% of BC patients are diagnosed at an early stage of the disease. We hypothesize that the improved survival observed in BC patients is partly because of this difference between Ardabil and BC regarding the distribution of disease stage. One of the reasons for lower survival rates in Ardabil province may be largely explained by differences in screening practices. In BC, a province-wide screening mammography program was established in 1988. Overall breast cancer survival rates were higher in BC because of screening mammography and the use of new adjuvant therapies following surgery for breast cancer [7,13-16].

Despite current evidence supporting population-based screening for breast cancer, there is no organized screening program for breast cancer in Iran. Lack of a screening program, low awareness of breast cancer, earlier age at diagnosis, structural barriers to accessing cancer treatment services, low income and lack of a usual source of care may all contribute to lower survival from breast cancer in Iran. We believe that early detection and better management using standard screening and treatment guidelines would contribute considerably to improving survival from breast cancer in these women. However, the introduction of a population-based breast cancer screening program or a change in the healthcare system may require additional information.

Various survival rates for breast cancer have been reported in the literature among different ethnic populations [7,17-23]. Asian American women are reported to have better survival from breast cancer as compared to other major ethnic minority groups [24]. South Asian women with breast cancer have inconsistent reports, with better survival than others in England [25] and California [26] but worse survival in BC [7].

Hislop reported [7] clear differences in breast cancer survival among Chinese, South Asian and the BC general populations. Survival was highest in Chinese women, which is interesting given that the proportions being screened were similar among the three ethnic groups. South Asian women, however, had the poorest survival. It was speculated that differences in treatment practices and possibly cancer biology affecting tumor progression may exist among these ethnic groups. It has been reported that Asian women with ductal carcinoma in situ of the breast were more likely to undergo mastectomy than lumpectomy [7,27]), a finding not seen in other groups.

Maskarinc [20] reported that ethnic variations in survival time among health plan members in Hawaii were not the result of different treatments, but primarily due to differences in early detection of breast cancer. When these investigators examined demographics, disease characteristics, co-morbidity, and treatment patterns, TNM stage was the strongest predictor of survival and explained the observed ethnic survival differences.

An earlier Hawaiian study by these investigators [28], however, had reported that socioeconomic status and marital status also affected survival in breast cancer patients. They proposed three possible explanations for the survival differences: utilization of health services, tolerance of treatment, and biologic variations in disease. Firstly, health services were not equally available to all residents. Many natives lived in remote areas with few health care providers, and cultural beliefs and practices (incompatible with the Western health system) discouraged them from accepting available health care. Secondly, ethnic differences in pre-existing conditions, such as diabetes, hypertension, obesity, and heart disease, gave some natives lower tolerance to surgery, chemotherapy, and radiotherapy, leading to shortened survival. Thirdly, there may be biologic differences at the genetic or molecular level resulting in more aggressive disease with faster progression in some native women as compared to the other ethnic groups. With a better understanding of the genetic characteristics of breast cancer, this explanation can be more fully explored.

Our study has several limitations, including the short follow-up period, giving only a very short-term view of prognosis; the lack of detailed clinical information in the cancer registries, such as staging and treatment information; and the small study population in Ardabil province, resulting in a relatively large variance. In addition, the limitations of this study include the shortcomings of Ardabil cancer registry as it is newly established. However, the Ardabil cancer registry covers the entire province. Data are collected from hospitals, pathology laboratories, diagnostic radiology clinics, outpatient public and private clinics, death certificate files and an annual health census. The number of breast cancer cases is low in Ardabil because the population is small and has a young age-structure. The relatively young population is particularly important in our comparison to BC because pre-menopausal breast cancer patients often have more aggressive disease than other women. Furthermore, Ardabil has one of the lowest breast cancer incidence rates in the world; gastric and esophageal cancers are the most-common forms of cancer in Ardabil. This might be the result of women's late age at menarche, reproductive behaviors and increased breast-feeding (as part of religious beliefs). Our analyses assumed that patients from both registries were still living after 1 year if no death was reported, although this assumption depends largely on the sources of information regarding deaths in the respective populations. In any population, it is difficult to know whether a patient's death is due to their breast cancer. This issue is particularly relevant in a comparison of populations with very different healthcare systems. Our analysis concerns the overall mortality of breast cancer patients, although the causes of death and the veracity of reported causes might be substantially different in Ardabil and BC. Although the cases were diagnosed in 2003, we could not extend the length of follow-up beyond one year because survival data was only available in BC to the end of 2004.

There is need for interventions that specifically target women with low education. Fear and embarrassment contribute to the lack of use of screening services and breast self examination. To better understand these issues, and how screening and treatment decisions are made, qualitative information should be collected from breast cancer patients and their health care providers.

Community intervention programs aimed at better understanding known risk factors of breast cancer, healthy lifestyle, and the importance of early detection are needed to help reduce deaths from breast cancer. These educational sessions should also provide information on how to get a mammogram and the steps to take following a diagnosis of breast cancer.

In both Ardabil province and BC, there is ongoing need for public education and awareness regarding early detection of breast cancer. This requires the use of communication media such as radio and television advertisements and programs for a mass education program. In addition to educating the public, it is also important to educate health care providers, especially those with whom are most likely to have contact. There is a need for establishment and implementation of screening programs for breast cancer, including regular breast examination, clinical breast exam and mammography, so that early lesions can be detected with a better survival and reduction of mortality. However, the introduction of a population-based breast cancer screening program or a change in the healthcare system may require additional information. Continued research into the causes of the disease, its prevention and improved methods of detection and treatment are essential if we are to make inroads into the control of this important cancer on a global scale.

## Conclusion

Breast cancer patients in BC had greater one-year survival rates than patients in Ardabil overall and for each age group under 60. These findings support the need for breast cancer screening programs (including regular clinical breast examinations and mammography), public education and awareness regarding early detection of breast cancer, and education of health care providers

## Competing interests

The authors declare that they have no competing interests.

## Authors' contributions

AS performed the statistical analysis and drafted the manuscript. TGH conceived of the study, and participated in its design and revision of the manuscript. CB conceived of the study, and participated in its design and statistical analysis. MB participated in design and statistical analysis. AG participated in coordination. MN participated in the design of the study. AG participated in data collection. RM conceived and supervised of the study. PY conceived of the study, and participated in its design, coordination, and drafted and revised the manuscript. All authors read and approved the final manuscript.

## Pre-publication history

The pre-publication history for this paper can be accessed here:

http://www.biomedcentral.com/1471-2407/9/381/prepub
